# Prevalence of Fluid Overload in a Cohort of Patients With Acute Pancreatitis: Results From a Retrospective Tertiary Single-Center Study

**DOI:** 10.1097/MPA.0000000000002534

**Published:** 2025-07-31

**Authors:** Nora Schneider, Maximilian Scharpf, Benjamin Mayer, Thomas Seufferlein, Alexander Kleger, Martin Müller, Viktoria Hentschel

**Affiliations:** * Department of Internal Medicine I, Ulm University Hospital; † Institute for Epidemiology and Medical Biometry, Ulm University; ‡ Institute for Molecular Oncology and Stem Cell Biology, Ulm University Hospital; § Division of Interdisciplinary Pancreatology, Department of Internal Medicine I, Ulm University Hospital

**Keywords:** acute pancreatitis, pancreatic fluid collection, fluid resuscitation

## Abstract

**Objectives:**

Aggressive fluid expansion has been an undisputed aspect of navigating the early stages of acute pancreatitis. Evidence produced by the WATERFALL trial supports a more conservative approach to fluid resuscitation as patients face higher odds of fluid overload while experiencing no benefit of their underlying condition. This retrospective monocentric observational study seeks to identify fluid-related complications in patients with acute pancreatitis of any grade of severity.

**Methods:**

One hundred twenty-nine patients with acute pancreatitis including moderately severe and severe courses were divided into groups based on their cumulative fluid administration within the first 24 hours (restrictive: <3 L; moderate: 3–6 L; aggressive: >6 L). Vital signs and laboratory values were reported at defined time points.

**Results:**

Most patients were allocated to the group of moderate fluid resuscitation. At baseline, patients did not differ in systemic disease severity indicated by critical illness scores. A higher proportion of patients with moderately severe or severe pancreatitis associated with an acute peripancreatic fluid collection had previously been administered >6 L fluids. C-reactive protein levels were significantly higher in patients with >6 L, whereas albumin levels were decreased.

Patients receiving >6 L fluids were more likely to show radiologic features of pulmonary fluid overload and be transferred to intensive care unit. In addition, oxygenation markedly deteriorated in aggressively hydrated patients versus restrictive and moderate fluid regimes.

**Conclusions:**

The extent of fluid resuscitation was found to be strongly associated with the prevalence of clinical and radiologic signs of pulmonary fluid overload. Furthermore, moderately severe and severe courses of pancreatitis were more frequently observed in excessively hydrated patients.

Acute pancreatitis (aP) remains a challenging condition associated with morbidity and occasional fatality.^1,2^ The underlying pathophysiology is characterized by excessive release of pro-inflammatory and vasoactive mediators from the inflamed pancreatic tissue. As a result, endothelial leakage causes a net fluid shift from the vasculature into serous cavities and tissue edema, promoting hemodynamic instability.^3^

Timely administration of crystalline fluids plays a crucial role in preventing hypovolemia and ensuring adequate organ perfusion. Historically, studies have supported a liberal stance towards fluid replacement, which has remained a cornerstone in the management of aP until nowadays. However, this viewpoint is increasingly being questioned by recent research, stressing potential adverse effects associated with excessive fluid administration, including the risk of pulmonary edema and pleural effusions, need for ventilatory support, and extended hospital stays.^4^ Owing to a lack of robust evidence, both national and international guidelines are inconsistent regarding the optimal amount of fluid administration in the management of aP, ranging between 130 and 500 mL/h.^5–7^ Collectively, international societies advocate a goal-directed approach, emphasizing the importance of hemodynamic monitoring to tailor fluid administration to individual patient needs.^5,8,9^ However, goal-directed fluid administration in an emergency setting may not always be feasible due to time constraints, limited staff availability, and the need for rapid assessment and stabilization of patients.^10^ Thus, general recommendations to regulate early fluid management are warranted.

Our study was inspired by the open-label WATERFALL trial, which aimed to assess the impact of moderate versus aggressive fluid resuscitation in patients with non-severe aP. Primary endpoint was the occurrence of moderately severe or severe pancreatitis during the hospital stay. However, early signs of fluid overload combined with lacking improvement of the disease course mandated premature termination of the trial during the first interim analysis.^11^ Therefore, it cannot be excluded that early cessation of the trial prevented achieving the primary endpoint due to insufficient statistical power. In addition, the incidence of moderate-to-severe fluid overload remained low in the WATERFALL trial, and a significant number of patients exhibited peripheral edema as the sole indicator of fluid overload. Ethical concerns related to exposing patients to potentially harmful fluid regimes can be mounted by retrospective data analysis from patients who have undergone resuscitation according to established institutional guidelines.

In this study, we aimed to build upon the findings of the WATERFALL trial by investigating clinical and radiologic signs of fluid overload across non-severe, moderately severe and severe pancreatitis, which might have previously been overlooked. We also focused on assessing the impact of fluid management on disease severity, systemic inflammatory response, length of inpatient hospital stays, and likelihood of requiring admission to the intensive care unit (ICU).

## MATERIALS AND METHODS

### Study Population

The study was designed as a retrospective monocentric study conducted at University Hospital Ulm, Germany. Three hundred fifty patients matching the International Classification of Disease (ICD) code “Acute pancreatitis, not further specified” (K85.x) hospitalized between 07/2020 and 09/2022 were screened for eligibility. To be enrolled in the study, patients needed to be diagnosed with aP according to the revised international consensus-based Atlanta classification^12^ and German national guidelines^13^ and to remain institutionalized for ≥48 hours. Exclusion criteria comprised patients not fulfilling above-mentioned diagnostic criteria of aP; in-patient treatment lasting <48 hours; referral from external health care facilities; pre-existing requirement for oxygen insufflation; aP not being the leading cause of hospitalization in patients with multiple medical conditions; incomplete documentation of clinical or laboratory records. Scrutinizing medical records yielded n=129 patients considered suitable for inclusion.

**FIGURE 1:**
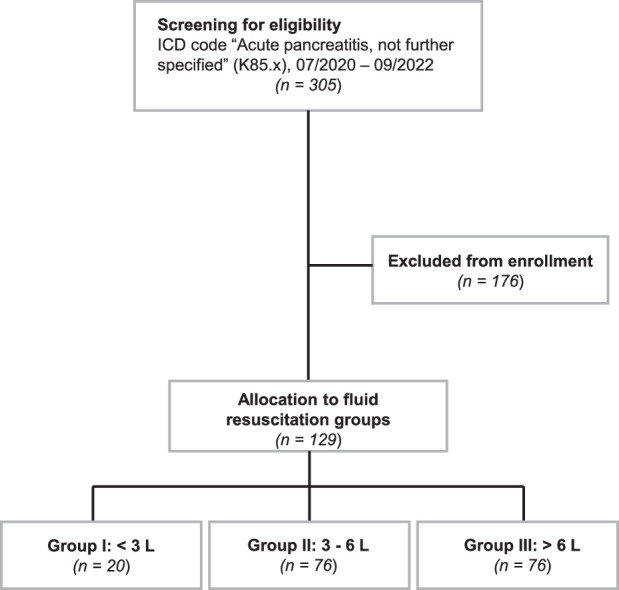
STROBE flow chart of documented study subjects with acute pancreatitis eligible for enrollment in this observational study. Allocation to groups was based on the total amount of fluids received within the first 24 hours from admission.

**FIGURE 2:**
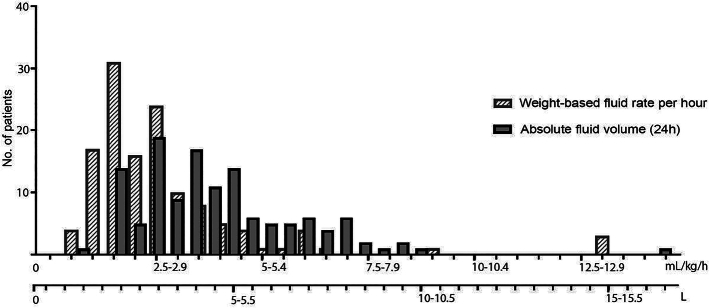
Spectra of fluid resuscitation regimes related to absolute fluid volume received within the first 24 hours (solid bars) and dosed per weight and hour (hatched bars).

On the basis of the fluid volume administered within the first 24 hours patients were grouped into 3 cohorts managed with restrictive (<3 L, n=20, group I), moderate (3–6 L, n=76, group II), or aggressive fluid (>6 L, n=33, group III) resuscitation (Fig. 1). The cutoffs applied to intersect the patient population were adapted from the weight-based calculations of non-aggressive versus aggressive fluid management used in the WATERFALL Trial,^11^ amounting to a cumulative volume of ~3 L in the non-aggressive and 6 L in the aggressive group in a 70 kg patient, respectively. Fluid replacement was generally guided by institutional protocols with an initial bolus of 10 mL/kg, followed by an infusion rate of 4 mL/kg/h (<24 h). Figure 2 depicts the distribution of patients based on the absolute fluid volume in 24 hours and weight-based fluid rates per hour.

### Data Collection

Vital signs and laboratory values were reported at defined time points (baseline, 48 hours, value_minimum_ or value_maximum_ within the first 72 hours). Patient charts were examined for documented symptoms of respiratory distress and objective signs indicative of pulmonary fluid overload upon physical examination. An abdominal compartment syndrome was defined by intra-abdominal pressure readings >20 mm Hg in conjunction with organ dysfunction.

As only few patients became dependent on advanced ventilatory support, concomitant monitoring of partial pressure of arterial oxygen (p_a_O_2_) was not available for most patients. For assessment of pulmonary function, we therefore resorted to imputing p_a_O_2_ values from oxygen saturation readings measured with pulse oximetry, using a nonlinear equation established from a data set of patients with ARDS^14^ to calculate the p_a_O2/ F_i_O_2_ ratio (hereinafter termed Horovitz ratio, HR). The inspiratory fraction of oxygen (F_i_O_2_) in patients with oxygen supply through nasal cannula or face mask was estimated from the respective flow rate using previously published conversion tables.^15^

In addition, patients with a relevant pulmonary fluid overload were identified by searching radiology reports for mentioning the following key features: Kerley lines, bilateral diffuse infiltrates (“bat wing pattern”), interstitial edema, peribronchial cuffing, ground-glass opacities, pleural effusions, dilated pulmonary veins, and prominent appearance of the upper vena cava.^16,17^

Clinical assessment was complemented by a defined set of laboratory parameters including white blood cell count (WBC), hematocrit, blood urea nitrogen (BUN), base deficit, lactate dehydrogenase (LDH), glucose, and calcium all of which pertain to the Ranson Criteria, which aids in predicting severity of aP and rating prognosis. Some of these parameters overlap with the revised Japanese Severity Score for Acute Pancreatitis (JSSP), which shows a similar prognostic accuracy but plays an inferior role outside Japan and regional Asian countries.^18^

### Statistics

All statistical computing was performed using the free software package R Studio (version 2023.12.1.402,^19^). Categorical variables were presented in absolute numbers including percentage values (n, %). Quantitative variables were expressed as median values and their interquartile range. Normality of data distribution was confirmed by Shapiro-Wilk test. Levene test was used to assess for homogeneity of variance between all groups. Normally distributed continuous variables were compared by means of 2-way ANOVA followed by multiple *t* tests. Quantitative variables not meeting criteria of normal distribution and ordinal variables were analyzed by the nonparametric Kruskal-Wallis test. For significant results, pairwise post hoc analysis was performed with Dunn test. Categorical variables were compared using Pearson χ^2^ test followed by Fisher's pairwise exact test. A 2-sided *P*-value <0.05 was interpreted as statistically significant. Variables for which previously mentioned statistical tests had demonstrated a significant difference between groups were used to fit univariate regression analysis models. Variables identified as significant predictor variables at a significance level of *P*≤0.1 were then returned to multivariate regression analysis.

## RESULTS

### Patient Cohort

Patient characteristics are listed in Table 1. Most patients (n=78, 60%) enrolled in this study were male. The median age of all patients was 55 (41–65) years. Patients undergoing restrictive fluid management were 61 (43–73) years of age, whereas patients with aggressive fluid administration were 48 (40–59) years of age. Measures of systemic illness expressed by the SOFA and SIRS scores were similar among groups at baseline. Only one fatality was reported in the group receiving moderate fluid replacement. In 101 (78%) patients, aP was attributable to a specific etiology, with gall stones (n=41, 32%), alcohol abuse (n=30, 23%), and medical interventions involving the pancreas (n=10, 7.8%) accounting for the most frequent causes. Fifty patients (39%) experiencing an episode of aP had underlying chronic pancreatitis.

**TABLE 1:** Baseline Characteristics of Study Subjects Classified by Cumulative Fluid Administration Within 24 Hours From Admission: Restrictive (<3 L), Moderate (3–6 L), Aggressive (>6 L) Fluid Resuscitation.

	Administered fluid volume within the first 24 hours				Post hoc test		
	<3 L (n=20)	3–6 L (n=76)	>6 L (n=33)	*P*	I vs. II	I vs. III	II vs. III
Age [median (IQR)]	61 (43–73)	57 (43–64)	48 (40–59)				
Sex							
Female	8 (40)	29 (38)	14 (42)				
Male	12 (60)	47 (62)	19 (58)				
Body mass index [median (IQR)]	25 (22–27)	27 (24–30)	30 (23–33)				
SOFA				0.49			
0–1	14 (70)	41 (54)	16 (49)				
≥2	4 (20)	27 (36)	13 (39)				
Not reported	2 (10)	8 (10)	4 (12)				
Severity of pancreatitis				0.003**	0.006**	<0.001***	0.087
Mild	18 (90)	43 (57)	12 (36)				
Moderately severe	2 (10)	24 (32)	17 (52)				
Severe	0 (0)	9 (12)	4 (12)				
Moderately severe or severe pancreatitis due to PFC	2 (10)	23 (30)	17 (51)	0.006**	0.09	0.003**	0.051
APFC	2 (10)	21 (28)	14 (42)				
other	0 (0)	2 (2)	3 (9)				
Mortality	0 (0)	1 (1)	0 (0)	>0.9			
Transfer to ICU	2 (10)	21 (28)	18 (55)	0.002**	0.14	0.001**	0.009**
Length of stay on ICU [median (IQR)]	2.0 (1.5–2.5)	3.0 (2.0–6.0)	3.0 (2.0–5.5)	0.6			
Length of in-patient stay [median (IQR)]	5.5 (4.0–7.0)	6.0 (4.0–10.3)	8.0 (6.0–11.0)	0.025*	0.2	0.008**	0.055
Etiology of pancreatitis							
Alcohol	5 (25)	14 (18)	11 (33)				
Gall stones	4 (20)	26 (34)	11 (33)				
Iatrogenic	2 (10)	4 (5)	4 (12)				
Drugs	0 (0)	2 (3)	0 (0)				
Autoimmune	0 (0)	2 (3)	1 (3)				
Hypertriglyceridemia	1 (5)	4 (5)	2 (6)				
Obstructive (other than gall stones)	1 (5)	1 (1)	1 (3)				
Unknown cause	7 (35)	23 (30)	3 (9)				
Substance abuse							
Alcohol	7 (35)	22 (29)	12 (36)				
Nicotine	9 (45)	35 (46)	18 (55)				
Comorbidities							
Cardiac	12 (60)	42 (55)	13 (39)				
Pulmonary	1 (5)	6 (8)	2 (6)				
Hepatic	9 (45)	29 (38)	11 (33)				
Metabolic	11 (55)	42 (55)	19 (58)				
Chronic kidney disease (KDIGO ≥2)	1 (5)	31 (41)	2 (6)				
Chronic pancreatitis	8 (40)	4 (5)	11 (33)				

Data are n (%) unless otherwise noted. Data were analyzed either by Kruskal-Wallis test or Pearson χ^2^ test followed by appropriate post hoc pairwise testing among groups. A *P*-value of <0.05 was considered statistically significant.

APFC indicates acute peripancreatic fluid collection; ICU, intensive care unit; IQR, interquartile range; PFC, pancreatic fluid collection.

### Excessive Fluid Resuscitation Fosters Clinical and Radiographic Features of Fluid Overload

In a first step, we focused on clinical signs and symptoms, typically indicative of fluid over-supply as a potential consequence from iatrogenic hyperhydration (Table 2). Overall, dyspnea was reported only by a small fraction of patients per group, which, despite being more prevalent in patients with higher fluid load, did not translate into significant differences between groups. Similarly, pulmonary rales were noted in 10 patients all of whom belonged to groups with moderate or aggressive fluid replacement. Similarly, peripheral edema was only present in patients with moderate or aggressive fluid resuscitation. An abdominal compartment syndrome was exclusively found in patients undergoing moderate or aggressive fluid resuscitation.

**TABLE 2:** Prevalence of Clinically and Radiologically Obvious Fluid-Related Complications Depending on the Amount of Fluid Resuscitation.

	Administered fluid volume within the first 24 hours				Post hoc		
	<3 L (n=20)	3–6 L (n=76)	>6 L (n=33)	*P*	I vs. II	I vs. III	II vs. III
Clinical signs of fluid overload							
Dyspnea	1 (5)	14 (18)	10 (30)	0.085			
Pulmonary rales	0 (0)	7 (9)	3 (9)	0.5			
Peripheral edema	0 (0)	11 (14)	7 (21)	0.075			
Ascites	3 (15)	18 (24)	11 (33)	0.3			
Inferior cava vein >2.5 cm	0 (0)	1 (1)	0 (0)	>0.9			
Intra-abdominal hypertension	0 (0)	7 (0)	4 (12)	0.3			
Intra-abdominal compartment syndrome	0 (0)	6 (8)	2 (6)	0.5			
New-onset atrial fibrillation	0 (0)	2 (3)	0 (0)	>0.9			
Radiologic signs of pulmonary fluid overload							
≥1 positive criteria	2 (10)	16 (21)	15 (45)	0.006**	0.3	0.014*	0.012*

Data are n (%) unless otherwise noted. Statistical analysis was performed with Pearson χ^2^ test followed by Fisher pairwise exact test among groups. A *P*-value of <0.05 was considered statistically significant.

Next, we reviewed chest radiograph reports for radiologic stigmas pointing to fluid overload. Patients receiving aggressive hydration were more likely to exhibit at least one radiographic feature of pulmonary fluid overload. With univariate analysis, only aggressive fluid replacement was confirmed as the determining factor for radiologic detection of excess pulmonary fluid (Table S1, Supplemental Digital Content 1, http://links.lww.com/MPA/B397). Pleural effusions and congestion of pulmonary veins were most frequently detected. None of the evaluated chest radiographs raised suspicion for acute respiratory distress syndrome (ARDS).

### Increased Fluid Supply Entails Impaired Oxygenation

Next, we set out to assess to which extent alveolar oxygenation was affected by different fluid resuscitation strategies. Although at baseline HR was identical between groups a drop was noted at 48 hours, which became more pronounced with increasing amounts of fluids. Within the first 72 hours, impaired oxygenation was more common in patients with aggressive fluid resuscitation compared with restrictive and moderate fluid regimes (Tables 3–5). Univariate analysis further revealed that the HR_minimum_ positively correlated with its baseline value but was negatively impacted by age, acute kidney injury (AKI), and aggressive fluid replacement (Table S2, Supplemental Digital Content 1, http://links.lww.com/MPA/B397). These significant relationships persisted when multivariate analysis was performed.

**TABLE 3:** Comparison of Results From Laboratory Monitoring Among Groups Treated With Different Fluid Resuscitation Regimes at Baseline.

Baseline							
	Administered fluid volume within the first 24 hours				Post hoc test		
	<3 L (n=20)	3–6 L (n=76)	>6 L (n=33)	*P*	I vs. II	I vs. III	II vs. III
Horovitz ratio (FiO_2_/p_a_O_2_)	431 (390–496)	431 (360–496)	431 (390–628)	0.12			
Basic metabolic panel							
Albumin (g/L)	42 (38–45)	43 (41–46)	41 (38–44)	0.2			
Bilirubin (μM)	10 (7–18)	12 (8–23)	14 (8–27)	0.5			
Base excess (mM)	0.0 (−0.5 to 1.4)	−0.3 (−2.0 to 0.9)	0.3 (−1.0 to 2.1)	0.3			
Blood sugar (mg/dL)	125 (101–145)	123 (109–155)	121 (107–165)	0.6			
BUN (mM)	5.1 (5.0–7.2)	4.8 (3.9–7.8)	7.1 (67–89)	0.3			
Calcium (mM)	2.3 (2.3–2.4)	2.3 (2.3–2.5)	2.3 (2.2–2.4)	0.3			
Creatinine (µM)	75 (60–94)	76 (61–99)	79 (67–89)	>0.9			
Lactate (mM)	1.6 (1.3–2.3)	1.9 (1.5–2.6)	2.0 (1.4–3.0)	0.3			
LDH (U/L)	210 (162–262)	204 (177–266)	201 (184–268)	0.8			
pH	7.40 (7.38–7.42)	7.38 (7.36–7.42)	7.38 (7.35–7.43)	0.3			
Inflammatory markers							
CRP	9 (4–36)	9 (3–31)	6 (2–19)	0.4			
Complete blood count							
Leukocytes (Giga/L)	8.9 (7.8–11.3)	10.8 (8.4–13.4)	11.3 (9.3–13.8)	0.2			
Hematocrit	0.41 (0.39–0.43)	0.42 (0.38–0.44)	0.40 (0.39–0.43)	>0.9			
Platelets (Giga/L)	215 (186–301)	232 (187–279)	236 (199–295)	0.6			

Statistical analysis was performed using either 2-way ANOVA or Kruskal-Wallis test followed by appropriate post hoc pairwise testing among groups. A *P*-value of <0.05 was considered statistically significant.

BUN indicates blood urea nitrogen; CRP, C-reactive protein; LDH, lactate dehydrogenase.

**TABLE 4:** Comparison of Results From Laboratory Monitoring Among Groups Treated With Different Fluid Resuscitation Regimes (48 Hours From Admission).

48 hours from admission							
	Administered fluid volume within the first 24 hours				Post hoc test		
	<3 L (n=20)	3–6 L (n=76)	>6 L (n=33)	*P*	I vs. II	I vs. III	II vs. III
Horovitz ratio (FiO_2_/ p_a_O_2_)	411 (360–431)	360 (274–431)	337 (293–411)	0.071			
Basic metabolic panel							
Albumin (g/L)	36 (43–42)	34 (31–37)	30 (27–32)	0.001**	0.081	<0.001***	0.005**
Bilirubin (µM)	9 (7–12)	15 (10–24)	17 (9–20)	0.093			
Base excess (mM/L)	0.4 (−0.9 to 1.9)	1.3 (−0.6 to 3.0)	2.6 (1.2–3.4)	0.07			
Blood sugar (mg/dL)	97 (91–119)	104 (84–138)	112 (87–149)	0.9			
BUN (mM)	3.9 (3.0–3.0)	4.4 (3.9–4.8)	4.8 (2.7–7.0)	0.4			
Calcium (mM)	2.3 (2.2–2.4)	2.2 (2.1–2.3)	2.1 (2.0–2.1)	<0.001***	0.036*	<0.001***	0.001**
Creatinine (µM)	73 (58–87)	69 (56–89)	64 (59–76)	0.6			
Lactate (mM)	1.8 (1.2–2.4)	1.3 (1.1–2.0)	1.3 (1.1–1.5)	0.6			
LDH (U/L)	290 (254–298)	214 (180–312)	214 (180–312)	0.2			
pH	7.39 (7.36–7.42)	7.41 (7.39–7.43)	7.41 (7.38–7.43)	0.6			
Inflammatory markers							
CRP (mg/L)	58 (4–162)	34 (31–37)	30 (27–32)	0.028*	0.4	0.020*	0.026*
Complete blood count							
Leukocytes (Giga/L)	7.2 (6.9–11.3)	8.1 (6.2–10.7)	9.4 (8.1–11.0)	0.2			
Hematocrit	0.39 (0.36–0.43)	0.37 (0.34–0.40)	0.35 (0.32–0.36)	0.06			
Platelets (Giga/L)	217 (173–267)	196 (165–215)	178 (131–221)	0.4			

Statistical analysis was performed using either 2-way ANOVA or Kruskal-Wallis test followed by appropriate post hoc pairwise testing among groups. A *P*-value of <0.05 was considered statistically significant.

BUN indicates blood urea nitrogen; CRP, C-reactive protein; LDH, lactate dehydrogenase.

**TABLE 5:** Comparison of Results From Laboratory Monitoring Among Groups With Different Fluid Resuscitation Regimes (Minimum or Maximum Readings Recorded Within 72 Hours From Admission).

Minimum/maximum readings (72 hours from admission)							
	Administered fluid volume within the first 24 hours				Post hoc test		
	<3 L (n=20)	3–6 L (n=76)	>6 L (n=33)	*P*	I vs. II	I vs. III	II vs. III
Temperature (°C) ↑	37.2 (37.0–37.8)	37.3 (37.0–37.6)	37.5 (37.3–38.1)	0.028*	0.9	0.073	0.009**
Heart rate (/min) ↑	97 (84–104)	93 (82–104)	98 (88–108)	0.3			
Mean arterial pressure (mm Hg) ↓	79 (74–87)	77 (72–83)	79 (73–85)	0.5			
Respiratory rate (/min) ↑	18 (15–22)	19 (18–25)	22 (18–27)	0.2			
Horovitz ratio (F_i_O_2_/ p_a_O_2_) ↓	360 (319–360)	337 (304–390)	285 (239–337)	0.2			
Basic metabolic panel							
Albumin (g/L) ↓	36 (34–44)	34 (30–42)	30 (27–33)	<0.001***	0.2	<0.001***	0.002**
Bilirubin (mM) ↑	11 (8–22)	16 (11–37)	21 (12–34)	0.074			
Base excess (mM/L) ↓	−0.2 (−1.5 to 0.6)	−0.3 (−2.5 to 0.8)	−0.7 (−2.8 to 0.5)	0.7			
Blood sugar (mg/dL) ↑	129 (108–168)	145 (118–173)	166 (123–217)	0.2			
BUN (mM)↑	5.1 (3.7–5.6)	5.5 (4.1–8.0)	4.7 (2.9–8.8)	0.6			
Calcium (mM)↓	2.2 (2.2–2.3)	2.1 (2.1–2.2)	2.0 (2.0–2.1)	<0.001***	0.040*	<0.001***	0.001**
Creatinine (µM)↑	79 (68–94)	80 (67–103)	81 (69–98)	0.2			
Lactate (mM)↑	1.7 (1.5–2.5)	2.0 (1.5–2.8)	2.1 (1.5–3.6)	0.4			
LDH (U/L)↑	218 (162–298)	217 (184–298)	300 (227–378)	0.011*	0.6	0.014*	0.006**
pH ↓	7.38 (7.36–7.40)	7.37 (7.35–7.40)	7.36 (7.33–7.39)	0.2			
Inflammatory markers							
CRP (mg/L) ↑	53 (6–118)	108 (18–241)	155 (74–298)	0.007**	0.062	0.002**	0.048*
Complete blood count							
Leukocytes (Giga/L) ↑	9.9 (8.6–13.0)	11.4 (9.0–14.9)	13.4 (10.4–16.1)	0.046*	0.3	0.020*	0.055
Hematocrit ↓	0.37 (0.36–0.42)	0.35 (0.32–0.38)	0.33 (0.31–0.35)	0.006**	0.0019**	0.001**	0.13
Platelets (Giga/L) ↓	183 (142–233)	179 (148–208)	174 (107–199)	0.5			

Statistical analysis was performed using either 2-way ANOVA or Kruskal-Wallis test followed by appropriate post hoc pairwise testing among groups. A *P*-value of <0.05 was considered statistically significant.

BUN indicates blood urea nitrogen; CRP, C-reactive protein; LDH, lactate dehydrogenase.

Aggressively hydrated patients faced the need for critical care medicine more often than patients with a more restrictive fluid management (Table 2). With univariate analysis, aggressive fluid replacement emerged as the only independent predictor of admission to ICU (Table S3, Supplemental Digital Content 1, http://links.lww.com/MPA/B397). Likewise, discharge from in-patient care was delayed in patients receiving aggressive fluid management compared with a restrictive fluid balance. Besides aggressive fluid replacement, AKI was identified as a positive predictor of prolonged hospitalization while male sex was of borderline significance (Table S4, Supplemental Digital Content 1, http://links.lww.com/MPA/B397). Only AKI retained its statistical significance in multivariate analysis.

### Pancreatic Fluid Collections Coincide With Aggressive Fluid Resuscitation

Several scoring systems exist to stratify patients with aP according to their risk of a severe disease course. The Revised Atlanta Classification differentiates between mild aP, moderately severe aP, and severe aP.^12^ In our study, moderately severe and severe courses of aP were more likely to be associated with aggressive versus restrictive fluid resuscitation. Similar observations were made when pancreatic fluid collections (PFC) of any type were considered (Table 1). In univariate analysis, the grade of aP was significantly influenced by moderate and aggressive fluid replacement, male sex, AKI, and disease etiology (Table S5, Supplemental Digital Content 1, http://links.lww.com/MPA/B397). Similarly, the frequency of PFC was found to be positively modulated by escalating fluid resuscitation and male sex of which only aggressive fluid regimes remained statistically significant in multivariate analysis (Table S6, Supplemental Digital Content 1, http://links.lww.com/MPA/B397).

### Aggressively Hydrated Patients Exhibit Higher Levels of Systemic Inflammation

Selected laboratory markers are shown in Tables 3–5.

Irrespective of the amount of fluids, baseline C-reactive protein (CRP) did not differ between groups. Both CRP at 48 hours and CRP_max_ were significantly elevated in patients with aggressive fluid replacement compared with moderate and restrictive fluid regimes, respectively. Besides aggressive fluid resuscitation, baseline CRP, male sex, and AKI were identified as independent variables with a significant impact on CRP at 48 hours (Table S7, Supplemental Digital Content 1, http://links.lww.com/MPA/B397). In multivariate analysis, only aggressive fluid resuscitation and baseline CRP were found to predict CRP levels. These findings were mirrored by WBC_max_, being significantly higher in aggressively versus restrictively hydrated patients.

In parallel to CRP, we tracked changes in albumin levels. At 48 hours, albumin successively declined with increasing fluid amounts, resulting in significant differences between aggressive and restrictive or moderate fluid resuscitation, respectively. Albumin_min_ showed an identical pattern. Albumin at 48 hours was negatively influenced by AKI, moderate and aggressive fluid resuscitation, all of which remained significant predictors in multivariate analysis (Table S8, Supplemental Digital Content 1, http://links.lww.com/MPA/B397).

LDH_max_ activity, which is reflective of the extent of pancreatic tissue necrosis, did not differ between restrictively and moderately hydrated patients but exhibited a surge in aggressively hydrated patients. Conversely, calcium_min_ and calcium at 48 hours dropped with rising fluid supply, resulting in a markeddifference between restrictively and aggressively hydrated patients.

Expectably, moderately and aggressively hydrated patients exhibited significantly lower levels of hematocrit_min_. Other parameters pertaining to the Ranson Criteria and JSSP which were covered by statistical analysis, were not significantly affected by the amount of fluid administration and therefore will not be explicitly reported in this section.

## DISCUSSION

Among numerous clinical studies, the WATERFALL trial stands out as one of the few prospective randomized trials focusing on the effects of aggressive fluid resuscitation as part of the standard care for aP. However, safety concerns regarding fluid overload led to its early termination during interim analysis after only 249 patients had been enrolled. Consequently, the trial’s primary outcome failed to demonstrate a meaningful impact of fluid volume on disease progression. The authors acknowledged a limitation in their study related to the a priori exclusion of patients at high risk for severe disease, which does not adequately represent the diverse patient population encountered in real-world practice.

We thus felt the need to contribute an adjunctive study from our tertiary referral center, reporting cases of both mild and severe disease. Notably, this study uncovers the prevalence of subtle clinical and overt radiologic signs of fluid overload across different fluid regimes. We observed a gradual increase in the prevalence of radiologic signs of pulmonary fluid overload, which became most pronounced in aggressively hydrated patients. This finding was corroborated on organ-function level, showing a relevant reduction in HR in response to heavy fluid load. Easily recognizable but oftentimes neglected clinical signs of generalized fluid overload tended to be more prevalent in moderately and aggressively hydrated patients. In line with that, a meta-analysis comparing the clinical outcomes between aggressive and non-aggressive hydration for aP revealed an increased risk of respiratory failure.^20^ From the pooled background of the meta-analysis, it remains debatable to which extent respiratory failure was caused by concomitant ARDS. Similar results were obtained from 2 retrospective studies.^21,22^

Although the WATERFALL trial indicated a trend towards higher rates of local complications such as pancreatic necrosis followingaggressive fluid resuscitation, our study identified a correlation between the amount of fluids and PFC prevalence, specifically APFC. Moreover, patients undergoing aggressive fluid resuscitation were more likely to be admitted to ICU and to endure longer hospital stays. These findings align with the results from the WATERFALL trial, which noted a weak positive relationship between fluid volume and indirect measures of disease severity. Furthermore, a previously published prospective cohort study from the same group found that administering more than 4.1 L/24 h was associated with respiratory insufficiency and PFC formation.^23^

We assessed laboratory markers reflecting the systemic inflammatory response at different time points. A significant increase in CRP levels was noted in aggressively hydrated patients, whereas albumin levels—most likely due a negative acute phase reaction rather than gross transendothelial leakage—showed an inverse time course. Endothelial dysfunction and systemic inflammation trigged by a positive fluid balance is a well-characterized phenomenon in patients with hemodialysis-dependent chronic kidney disease.^24^ It is therefore conceivable that the fluid overload may have fueled the inflammatory response we observed in the aggressively hydrated group. Also, we noticed that male sex heralds a higher risk of developing a PFC and a systemic inflammatory response; this finding is supported by a large retrospective study based on the national inpatient database 2016–2017, which revealed a lower risk forfemale pancreatitis patients to beadmitted to ICU or requirepancreatic drainage.^25^ Meanwhile, oxygenating capacity was the only clinical outcome found to be negatively impacted by age, which may be explained by higher odds of cardiac or pulmonary impairment in elderly patients. Interestingly, as opposed to alcohol consumption, gall stones as another main inciting event of aP were less likely to entail complicated disease phenotypes including formation of PFC which is in line with previous studies.^26,27^

With this study, we aim at shedding some light on the sequelae of excessive fluid therapy in the management of aP regarding adverse events and disease severity. As a tertiary referral center for gastrointestinal diseases, we hold medical records of a multitude of patients with aP of which only patients with complete data sets were considered for analysis. Unlike previous studies,^11,20,23^ we included laboratory markers of systemic inflammation recorded at different time points to underpin the clinical outcomes. Also, instead of choosing a dichotomous classification into restrictive versus aggressive fluid management we have diversified analysis by including a group of moderately hydrated patients.

However, we are aware that due to its retrospective design results are diluted by several limitations: firstly, we could ascertain imbalances in composition of groups, with patients undergoing a restrictive fluid regime being older and more likely to be diagnosed with cardiac and hepatic disorders, both of which are notoriously prone to fluid retention. Secondly, although statistics yielded no significant differences in baseline disease severity across groups, we cannot exclude that patients whose clinical appearance prompted physicians to anticipate a more complicated disease course may have been exposed to higher amounts of fluids from the beginning. Therefore, it cannot be determined with certainty to what extent inflammation was promoted by the fluid challenge or whether patients expected to be more severely diseased were more extensively hydrated. Interestingly, despite their advanced age and relevant comorbidities, restrictively hydrated patients exhibited the most favorable outcome among all groups, although aP in elderly patients portends a worse overall prognosis.^28^ This seemingly contradictory finding might indicate that restrictive fluid supply may compensate for relevant risk factors, thus allowing the indirect conclusion that the extent of fluid resuscitation affects the severity of pancreatitis.

Our results align with previous results from the WATERFALL trial, showing an increased risk of iatrogenic fluid overload in excessively hydrated patients with aP. Furthermore, we demonstrate that aggressive fluid resuscitation is associated with moderately severe or severe aP, prolonged hospitalization, and need for intensive care. Although the retrospective design does not permit conclusions on causal relationships, results imply that excessive fluid resuscitation may additionally aggravate pancreatic inflammation. Further investigation on the appropriate fluid management in patients with moderate and severe aP is required.

## Supplementary Material

SUPPLEMENTARY MATERIAL
