# Supplementary material for: Prevalence of Fluid Overload in a Cohort of Patients With Acute Pancreatitis: Results From a Retrospective Tertiary Single-Center Study
Source: Pancreas. 2025 Jul 31;55(2):e212–9. doi: 10.1097/MPA.0000000000002534 (PMC12788752; doi:10.1097/MPA.0000000000002534)
Supplement: SUPPLEMENTARY MATERIAL [file mpa-55-e212-s001.docx]

| **Radiological signs of pulmonary fluid overload** | | | | | | |
| --- | --- | --- | --- | --- | --- | --- |
|  | **Univariate** | | | **Multivariate** | | |
|  | Beta | 95% CI | *P* value | Beta | 95% CI | *P* value |
| **Male sex** | 1.20 | 0.53; 2.77 | 0.7 | - | - | - |
| **Age** | 1.00 | 0.97; 1.02 | >0.9 | - | - | - |
| **BMI** | 1.00 | 0.93; 1.06 | >0.9 | - | - | - |
| **Baseline SOFA ≥ 2** | 0.98 | 0.40; 2.31 | >0.9 | - | - | - |
| **Biliary etiology of aP** | 0.49 | 0.18; 1.20 | 0.14 | - | - | - |
| **Fluid administered**   - Moderate (3-6 L) - Excessive (> 6 L) | 2.40  7.50 | 0.60; 16.1  1.77; 52.1 | 0.3  **0.014** | -  - | -  - | -  - |
| **General comorbidities**   - Cardiac - Pulmonary - Hepatic - Metabolic | 1.60  2.51  1.28  1.10 | 0.72; 3.65  0.59; 10.1  0.57; 2.87  0.50; 2.48 | 0.2  0.2  0.5  0.8 | -  -  -  - | -  -  -  - | -  -  -  - |
| **Specific comorbidities**   - Chronic kidney disease (KDIGO ≥ 2) - Acute kidney injury - Chronic pancreatitis | 0.47  2.68  1.04 | 0.02; 2.89  0.72; 9.56  0.45; 2.32 | 0.5  0.13  >0.9 | -  -  - | -  -  - | -  -  - |

**Table S1.** Relationship between the presence of radiological features of pulmonary fluid overload (dependent variable) and potential predictor variables (independent variables).

Statistical analysis by univariate regression analysis, *P* ≤ 0.1. Multivariate regression analysis not applicable due to output of significant independent variables < 2.

BMI, body mass index; SOFA, sequential organ failure assessment; aP, acute pancreatitis; KDIGO, Kidney Disease Improving Global Outcomes.

| **Horovitz ratio_min_ (72h from admission)** | | | | | | |
| --- | --- | --- | --- | --- | --- | --- |
|  | **Univariate** | | | **Multivariate** | | |
|  | Beta | 95% CI | *P* value | Beta | 95% CI | *P* value |
| **Male sex** | -13 | -47; 21 | 0.5 | - | - | - |
| **Age** | -1.7 | -2.7; -0.70 | **<0.001** | -1.4 | -2.4; -0.38 | **0.007** |
| **BMI** | 1.0 | -1.6; 3.6 | 0.4 | - | - | - |
| **Baseline SOFA ≥ 2** | -31 | -67; 4.4 | **0.085** | 2.4 | -32; 37 | 0.9 |
| **Biliary etiology of aP** | -0.23 | -36; 35 | > 0.9 | - | - | **-** |
| **Baseline Horovitz ratio** (F_i_O_2_/p_a_O_2_) | 0.26 | 0.09; 0.42 | **0.002** | 0.24 | 0.07; 0.41 | **0.007** |
| **Fluid administered**   - Moderate (3-6 L) - Excessive (> 6 L) | -5.3  -55 | -52; 42  -109; -2.2 | 0.8  **0.041** | -0.55  -63 | -46; 45  -115; -11 | >0.9  **0.018** |
| **General comorbidities**   - Cardiac - Pulmonary - Hepatic - Metabolic | -23  -31  -8.8  -1.9 | -56; 9.9  -96; 34  -43; 25  -36; 32 | 0.2  0.3  0.6  >0.9 | -  -  -  - | -  -  -  - | -  -  -  - |
| **Specific comorbidities**   - Chronic kidney disease (KDIGO ≥ 2) - Acute kidney injury - Chronic pancreatitis | -27  -76  11 | -100; 46  -134; -18  -23; 46 | 0.5  **0.010**  0.5 | -  -66  - | -  -122; -10  - | -  **0.021**  - |

**Table S2.** Relationship between the minimum Horovitz ratio within the first 72 hours (Horovitz_min_, dependent variable) and potential predictor variables (independent variables).

Statistical analysis by univariate regression analysis (*P* value ≤ 0.1). Any independent variables tested statistically significant were subject to multivariate regression analysis. A *P* value of ≤ 0.05 was considered significant.

BMI, body mass index; SOFA, sequential organ failure assessment; aP, acute pancreatitis; KDIGO, Kidney Disease Improving Global Outcomes.

| **Transfer to ICU** | | | | | | |
| --- | --- | --- | --- | --- | --- | --- |
|  | **Univariate** | | | **Multivariate** | | |
|  | Beta | 95% CI | *P* value | Beta | 95% CI | *P* value |
| **Male sex** | 1.20 | 0.56; 2.62 | 0.6 | - | - | - |
| **Age** | 1.00 | 0.98; 1.02 | >0.9 | - | - | - |
| **BMI** | 1.01 | 0.96; 1.07 | 0.6 | - | - | - |
| **Baseline SOFA ≥ 2** | 1.85 | 0.82; 4.19 | 0.13 | - | - | - |
| **Biliary etiology of aP** | 0.71 | 0.30; 1.58 | 0.4 | - | - | - |
| **Fluid administered**   - Moderate (3-6 L) - Excessive (> 6 L) | 3.44  10.8 | 0.89; 22.8  2.56; 75.1 | 0.12  **0.004** | -  - | -  - | -  - |
| **General comorbidities**   - Cardiac - Pulmonary - Hepatic - Metabolic | 0.96  1.79  0.92  1.18 | 0.46; 2.02  0.42; 7.16  0.42; 1.96  0.56; 2.52 | >0.9  0.4  0.8  0.7 | -  -  -  - | -  -  -  - | -  -  -  - |
| **Specific comorbidities**   - Chronic kidney disease (KDIGO ≥ 2) - Acute kidney injury - Chronic pancreatitis | 0.85  2.85  0.75 | 0.12; 4.14  0.81; 10.5  0.34; 1.61 | 0.9  0.10  0.5 | -  -  - | -  -  - | -  -  - |

**Table S3.** Relationship between the likelihood of transfer to ICU (dependent variable) and potential predictor variables (independent variables).

Statistical analysis by univariate regression analysis, *P* ≤ 0.1. Multivariate regression analysis not applicable due to output of significant independent variables < 2.

ICU, intensive care unit; BMI, body mass index; SOFA, sequential organ failure assessment; aP, acute pancreatitis; KDIGO, Kidney Disease Improving Global Outcomes.

| **Length of hospital stay** | | | | | | |
| --- | --- | --- | --- | --- | --- | --- |
|  | **Univariate** | | | **Multivariate** | | |
|  | Beta | 95% CI | *P* value | Beta | 95% CI | *P* value |
| **Male sex** | 2.7 | -0.53; 6.0 | **0.10** | 1.1 | -1.8; 4.1 | 0.4 |
| **Age** | 0.07 | -0.03; 0.17 | 0.2 | - | - | - |
| **BMI** | -0.08 | -0,34; 0.17 | 0.5 | - | - | - |
| **Baseline SOFA ≥ 2** | 4.4 | 1.2; 7.6 | **0.008** | 1.8 | -1.3; 4.9 | 0.2 |
| **Biliary etiology of aP** | -0.85 | -4.3; 2.6 | 0.6 | - | - | - |
| **Fluid administered**   - Moderate (3-6 L) - Excessive (> 6 L) | 2.1  4.3 | -2.5; 6.7  -0.83; 9.5 | 0.4  **0.10** | -0.37  2.2 | -4.5; 3.7  -2.5; 6.8 | 0.9  0.4 |
| **General comorbidities**   - Cardiac - Pulmonary - Hepatic - Metabolic | 2.4  2.4  -1.8  -1.6 | -0.86; 5.6  -3.9; 8.7  -5.1; 1.5  -4.9; 1.6 | 0.15  0.5  0.3  0.3 | -  -  -  - | -  -  -  - | -  -  -  - |
| **Specific comorbidities**   - Chronic kidney disease (KDIGO ≥ 2) - Acute kidney injury - Chronic pancreatitis | 1.1  14  -0.58 | -6.0; 8.3  8,6; 19  -3.9; 2.7 | 0.8  **<0.001**  0.7 | -  13  - | -  7.8; 18  - | -  **<0.001**  - |

**Table S4.** Relationship between the length of hospital stay (dependent variable) and potential predictor variables (independent variables).

Statistical analysis by univariate regression analysis (*P* value ≤ 0.1). Any independent variables tested statistically significant were subject to multivariate regression analysis. A *P* value of ≤ 0.05 was considered significant.

BMI, body mass index; SOFA, sequential organ failure assessment; aP, acute pancreatitis; KDIGO, Kidney Disease Improving Global Outcomes.

| **Severe pancreatitis according to Atlanta classification** | | | | | | |
| --- | --- | --- | --- | --- | --- | --- |
|  | **Univariate** | | | **Multivariate** | | |
|  | Beta | 95% CI | *P* value | Beta | 95% CI | *P* value |
| **Male sex** | 2.19 | 1.07; 4.61 | **0.034** | - | - | - |
| **Age** | 1.01 | 0.99; 1.04 | 0.2 | - | - | - |
| **BMI** | 0.99 | 0.94; 1.04 | 0.7 | - | - | - |
| **Baseline SOFA ≥ 2** | 1.42 | 0.68; 2.96 | 0.3 | - | - | - |
| **Biliary etiology of aP** | 0.57 | 0.26; 1.20 | 0.15 | - | - | - |
| **Fluid administered**   - Moderate (3-6 L) - Excessive (> 6 L) | 7.36  14.0 | 1.95; 48.3  3.40; 96.0 | **0.010**  **0.001** | -  - | -  - | -  - |
| **General comorbidities**   - Cardiac - Pulmonary - Hepatic - Metabolic | 1.54  1.32  1.03  0.86 | 0.78; 3.07  0.31; 5.14  0.51; 2.06  0.44; 1.71 | 0.2  0.7  >0.9  0.7 | -  -  -  - | -  -  -  - | -  -  -  - |
| **Specific comorbidities**   - Chronic kidney disease (KDIGO ≥ 2) - Acute kidney injury - Chronic pancreatitis | 1.72  6.68  1.29 | 0.39; 7.23  2.13; 22.0  0.65; 2.57 | 0.5  **0.001**  0.5 | -  -  - | -  -  - | -  -  - |

**Table S5.** Relationship between the Atlanta score of pancreatitis severity (dependent variable) and potential predictor variables (independent variables).

Statistical analysis by univariate regression analysis, *P* ≤ 0.1. Multivariate regression analysis not permitted due to number of events < 10 per predictor variable among patients with severe pancreatitis.

BMI, body mass index; SOFA, sequential organ failure assessment; aP, acute pancreatitis; KDIGO, Kidney Disease Improving Global Outcomes.

| **Occurrence of PFC** | | | | | | |
| --- | --- | --- | --- | --- | --- | --- |
|  | **Univariate** | | | **Multivariate** | | |
|  | Beta | 95% CI | *P* value | Beta | 95% CI | *P* value |
| **Male sex** | 2.03 | 0.94; 4.61 | **0.079** | 1.91 | 0.83; 4.62 | 0.14 |
| **Age** | 0.99 | 0.97; 1.01 | 0.4 | - | - | - |
| **BMI** | 0.97 | 0.91; 1.03 | 0.3 | - | - | - |
| **Baseline SOFA ≥ 2** | 0.86 | 0.38; 1.90 | 0.7 | - | - | - |
| **Biliary etiology of aP** | 0.47 | 0.19; 1.07 | **0.083** | 0.46 | 0.18; 1.11 | 0.094 |
| **Fluid administered**   - Moderate (3-6 L) - Excessive (> 6 L) | 3.91  9.56 | 1.01; 25.8  2.27; 66.5 | **0.083**  **0.006** | 4.50  11.8 | 1.14; 30.2  2.70; 84,7 | 0.059  **0.003** |
| **General comorbidities**   - Cardiac - Pulmonary - Hepatic - Metabolic | 1.03  0.57  0.64  1.25 | 0.49; 2.16  0.08; 2.49  0.28; 1.37  0.60; 2.67 | >0.9  0.5  0.3  0.6 | -  -  -  - | -  -  -  - | -  -  -  - |
| **Specific comorbidities**   - Chronic kidney disease (KDIGO ≥ 2) - Acute kidney injury - Chronic pancreatitis | 0.82  2.73  1.49 | 0.11; 3.99  0.78; 10.0  0.70; 3.16 | 0.8  0.11  0.3 | -  -  - | -  -  - | -  -  - |

**Table S6.** Relationship between the occurrence of PFC (dependent variable) and potential predictor variables (independent variables).

Statistical analysis by univariate regression analysis (*P* value ≤ 0.1). Any independent variables tested statistically significant were subject to multivariate regression analysis. A *P* value of ≤ 0.05 was considered significant.

PFC, pancreatic fluid collection; BMI, body mass index; SOFA, sequential organ failure assessment; aP, acute pancreatitis; KDIGO, Kidney Disease Improving Global Outcomes.

| **Serum CRP (48h from admission)** | | | | | | |
| --- | --- | --- | --- | --- | --- | --- |
|  | **Univariate** | | | **Multivariate** | | |
|  | Beta | 95% CI | *P* value | Beta | 95% CI | *P* value |
| **Male sex** | 42 | -3.2; 87 | **0.068** | 32 | -9.2; 74 | 0.13 |
| **Age** | 0.74 | -0,63; 2.1 | 0.3 | - | - | - |
| **BMI** | 2.3 | -1.3; 6.0 | 0.2 | - | - | - |
| **Baseline SOFA ≥ 2** | 0.82 | -47; 49 | >0.9 | - | - | - |
| **Biliary etiology of aP** | -38 | -85; 8.2 | 0.11 | - | - | **-** |
| **Baseline serum CRP** (g/L) | 0.60 | 0.32; 0.89 | **<0.001** | 0.59 | 0.28; 0.89 | **<0.001** |
| **Fluid administered**   - Moderate (3-6 L) - Excessive (> 6 L) | 20  80 | -45; 85  9.5; 151 | 0.5  **0.026** | 19  87 | -40; 79  21; 153 | 0.5  **0.010** |
| **General comorbidities**   - Cardiac - Pulmonary - Hepatic - Metabolic | 22  -21  -8.8  15 | -23; 66  -106; 65  -54; 37  -30; 59 | 0.3  0.6  0.7  0.5 | -  -  -  - | -  -  -  - | -  -  -  - |
| **Specific comorbidities**   - Chronic kidney disease (KDIGO ≥ 2) - Acute kidney injury - Chronic pancreatitis | 6.4  79  -8.1 | -91; 104  3.0; 154  -54; 38 | 0.9  **0.042**  0.7 | -  -2.1  - | -  -79; 75  - | -  >0.9  - |

**Table S7.** Relationship between the serum CRP level (dependent variable) and potential predictor variables (independent variables).

Statistical analysis by univariate regression analysis (*P* value ≤ 0.1). Any independent variables tested statistically significant were subject to multivariate regression analysis. A *P* value of ≤ 0.05 was considered significant.

CRP, C-reactive protein; BMI, body mass index; SOFA, sequential organ failure assessment; aP, acute pancreatitis; KDIGO, Kidney Disease Improving Global Outcomes.

| **Serum albumin (48h from admission)** | | | | | | |
| --- | --- | --- | --- | --- | --- | --- |
|  | **Univariate** | | | **Multivariate** | | |
|  | Beta | 95% CI | *P* value | Beta | 95% CI | *P* value |
| **Male sex** | 0.13 | -2.8; 3.1 | >0.9 | - | - | - |
| **Age** | 0.08 | 0.00; 0.16 | **0.048** | 0.05 | -0.01; 0.12 | 0.12 |
| **BMI** | 0.04 | -0.20; 0.28 | 0.7 | - | - | - |
| **Baseline SOFA ≥ 2** | -4.1 | -6.9; -1.3 | **0.005** | -3.1 | -5.6; -0.56 | **0.018** |
| **Biliary etiology of aP** | 0.21 | -2.8; 3.3 | 0.9 | - | - | **-** |
| **Baseline serum albumin** (g/dL) | 0.61 | 0.34; 0.88 | **<0.001** | 0.45 | 0.19; 0.70 | **<0.001** |
| **Fluid administered**   - Moderate (3-6 L) - Excessive (> 6 L) | -3.8  -7.7 | -8.1; 0.49  -12; -3.3 | **0.081**  **<0.001** | -2.6  -5.2 | -6.5; 1.3  -9.2; -1.3 | 0.2  **0.011** |
| **General comorbidities**   - Cardiac - Pulmonary - Hepatic - Metabolic | 1.2  -1.6  0.06  2.9 | -1.6; 3.9  -6.9; 3.7  -2.8; 2.9  0.10; 5.6 | 0.4  0.5  >0.9  **0.043** | -  -  -  2.2 | -  -  -  -0.17; 4.5 | -  -  -  0.0068 |
| **Specific comorbidities**   - Chronic Kidney Disease (KDIGO ≥ 2) - Acute Kidney Injury - Chronic Pancreatitis | -0.18  -5.5  1.2 | -5.1; 4.7  -9,6; -1.4  -1,7; 4.0 | >0.9  **0.009**  0.4 | -  -2.7  - | -  -6.1; 0.68  - | -  0.11  - |

**Table S8.** Relationship between the serum albumin level (dependent variable) and potential predictor variables (independent variables).

Statistical analysis by univariate regression analysis (*P* value ≤ 0.1). Any independent variables tested statistically significant were subject to multivariate regression analysis. A *P* value of ≤ 0.05 was considered significant.

BMI, body mass index; SOFA, sequential organ failure assessment; aP, acute pancreatitis; KDIGO, Kidney Disease Improving Global Outcomes.
